# Association Analysis of Essential Tremor-Associated Genetic Variants in Sporadic Late-Onset Parkinson’s Disease

**DOI:** 10.5334/tohm.885

**Published:** 2024-05-10

**Authors:** Sheng Zeng, Xun Zhou, Runcheng He, Yuwen Zhao, Zhenhua Liu, Qian Xu, Jifeng Guo, Xinxiang Yan, Jinchen Li, Beisha Tang, Qiying Sun

**Affiliations:** 1 Department of Geriatrics, The Second Xiangya Hospital, Central South University, Changsha, Hunan 410011, China; 2 Key Laboratory of Hunan Province in Neurodegenerative Disorders, Central South University, Changsha, Hunan, 410008, China; 3 Department of Geriatric Neurology, Xiangya Hospital, Central South University, Changsha, Hunan 410008, China; 4 Department of Neurology, Xiangya Hospital, Central South University, Changsha, Hunan, 410008, China; 5 National Clinical Research Center for Geriatric Disorders, Xiangya Hospital, Central South University, Changsha, Hunan, 410008, China; 6 Center for Medical Genetics, School of Life Sciences, Central South University, Changsha, Hunan, 410008, China; 7 The Institute of Molecular Precision Medicine and Hunan Key Laboratory of Molecular Precision Medicine, Xiangya Hospital, Central South University, Changsha, China

**Keywords:** genetic variant, association analysis, rare deleterious variants, SKAT-O, Parkinson’s disease, essential tremor

## Abstract

**Background:**

Parkinson’s disease (PD) and Essential tremor (ET) are the two most common tremor diseases with recognized genetic pathogenesis. The overlapping clinical features suggest they may share genetic predispositions. Our previous study systematically investigated the association between rare coding variants in ET-associated genes and early-onset PD (EOPD), and found the suggestive association between *teneurin transmembrane protein 4* (*TENM4*) and EOPD. In the current research, we explored the potential genetic interplay between ET-associated genetic loci/genes and sporadic late-onset PD (LOPD).

**Methods:**

We performed whole-genome sequencing in the 1962 sporadic LOPD cases and 1279 controls from mainland China. We first used logistic regression analysis to test the top 16 SNPs identified by the ET genome-wide association study for the association between ET and LOPD. Then we applied the optimized sequence kernel association testing to explore the rare variant burden of 33 ET-associated genes in this cohort.

**Results:**

We did not observe a significant association between the included SNPs with LOPD. We also did not discover a significant burden of rare deleterious variants of ET-associated genes in association with LOPD risk.

**Conclusion:**

Our results do not support the role of ET-associated genetic loci and variants in LOPD.

**Highlights:**

1. 1962 cases and 1279 controls were recruited to study the potential genetic interplay between ET-associated genetic loci/variants and sporadic LOPD.
2. No significant association between the ET-associated SNPs and LOPD were observed.
3. No significant burden of rare deleterious variants of ET-associated gene in LOPD risk were found.

## Introduction

PD is a complex movement disorder in which genetics plays a remarkable role in its etiology [1]. With the emergence and popularity of sequencing technology, the genetic study of PD has achieved more rapid development and progress than ever before. Although our understanding of the genetic architecture of PD has expanded considerably, much remains to be done. Generally, genetic risk variants resulting in PD are often divided into two categories: rare variants with high effect sizes are more found in familial PD, the common variants with more minor effects are usually associated with apparently sporadic PD [2,3]. Additionally, genetic factors are important in both early-onset (EOPD, age at onset (AAO) ≦ 50 years) and late-onset (LOPD, AAO > 50 years) PD, but specific genes and mode of inheritance may differ between the two groups [4].

There has been a longstanding controversy surrounding the possible link between PD and another movement disorder, ET [5,6,7]. The two clinical entities have countless ties regarding their clinical, epidemiologic, imaging, pathologic, and genetic features [8,9,10,11,12]. Evidence from genetic research suggested that *LINGO1, LINGO2, HS1BP3, DNAJC13, HTRA2, NAACP-Rep1*, and *CACNA1G* were overlapping genetic risks between them [13]. A recent published genome-wide association study (GWAS) of ET revealed significant common variant overlap with PD [14]. Our previous study systematically investigated the association between rare coding variants in ET-associated genes and EOPD, and found the suggestive association between *teneurin transmembrane protein 4* (*TENM4)* and EOPD, which provided evidence for a genetic link between ET and PD [15]. In the current research, we explored the potential genetic interplay between ET-associated genetic loci/genes and LOPD from mainland China.

## Materials and Methods

### Patients and controls

1962 sporadic late-onset (AAO > 50 years) PD patients (mean AAO, 61.88 ± 6.93 years; mean age, 66.76 ± 7.08 years; 50.15% male) and 1279 race-matched healthy controls (mean age, 62.32 ± 7.11 years; 47.93% male) were included in this case-control study (Supplementary Table 1). Patients were recruited from the outpatient neurology clinics of Xiangya Hospital of Central South University from October 2006 to January 2019 and other cooperating centers of Parkinson’s Disease & Movement Disorders Multicenter Database and Collaborative Network in China (PD-MDCNC, http://pd-mdcnc.com/). All the patients were subjected to the standard clinical evaluation by at least two neurologists and diagnosed with PD according to the criteria from UK PD Brain Bank [16] or Movement Disorders Society (MDS) [17]. Patients with a positive family history of PD and other neurological diseases were excluded. The clinical subtypes of PD were classified as tremor-dominant (TD), postural instability and gait difficulty-dominant (PIGD), and intermediate types based on the ratio of the mean tremor score of the unified Parkinson’s disease rating scale (UPDRS) to the mean PIGD score [18], of which 453 cases (23.1%) were classified as tremor-dominant. Controls collected from the community did not have any neurological or psychiatric system diseases. Informed consent was obtained from all subjects, as approved by the Ethical Committee of Xiangya Hospital of the Central South University in China (equivalent to an Institutional Review Board). After obtaining informed consent, blood samples were obtained from the subjects described above. Genomic DNA was extracted from peripheral blood using standard extraction methods.

### Whole-genome sequencing (WGS) and bioinformatics pipeline

According to the manufacturer’s instructions, genomic DNA samples of subjects were used for library construction and followed by sequencing with Illumina NovaSeq platform (Illumina, San Diego, CA). Methods for the further bioinformatic pipeline were described previously [19,20]. Raw data obtained became clean data through quality control by FastQC (version 0.11.2, Andrews.2010) and was then aligned to the human genome 19 reference by Burrows-Wheeler Alignment Maximal Exact Matches algorithm (BWA-MEM) (version 0.7.12-r1044, Li.2010). SNVs and InDels were called from aligned BAM files using the Genome Analysis Toolkit-GATK v2.3–9. Variants were further annotated with ANNOVAR [21] and VarCards [22] based on RefSeq (UCSC hg19). Exclusion criteria for individuals and variants was conducted through PLINK1.90. Individuals were removed for gender discrepancy, deviating heterozygosity/genotype calls (> 3 standard deviations [SDs]) and cryptic relatedness measured using identity-by-descent (IBD > 0.15). Genotypes with a Phred-scaled genotype quality score (GQ) below 15 (for SNVs) or 30 (for InDels), allele depth below two, or reads depth below five were removed in our analysis. Variants were filtered for missing genotype rate (< 0.1) and Hardy-Weinberg equilibrium in controls (P > 0.0001) for the targeted regions. Principal component (PC) analysis on population stratification was performed in independent high-quality variants, and main PC variables for each sample were obtained for subsequent association analysis.

### Rare Variant selection from targeted gene

A set of ET-associated genes/loci included in our analysis (Supplementary Table 2). [15]. The East Asian population evaluated the minor allele frequencies (MAF) from the public database (ExAC and gnomAD database) [23] to determine rare variants (MAF < 1%). The exon-coding rare variants extracted from the targeted gene were included in our analysis. Our study defined damaging missense variants based on variant prediction by ReVe (> 0.7) [24]. Loss-of-function (LoF) variants were annotated as stop-gain/loss, frameshift, or splicing variants.

### Common variants selection from identified ET-GWAS study

ET genome-wide association study (ET-GWAS) has identified many putative ET loci, however, most of them had conflicting replication results. Five ET-GWAS papers were published and a total of 16 SNPs were found most significantly associated with ET until now (Table 1) [14,25,26,27,28]. All of the 16 SNPs were included in our analysis. Specifically, four of the 16 variants were newly identified loci from a large European case-control study [14]. Variants were excluded for departure from Hardy-Weinberg equilibrium among controls (P < 0.0001).

**Table 1:** Summary of SNPs associated with ET selected in our study.


SNP	GENE/LOCUS	POSITION (hg19)	MINOR ALLELE	MAJOR ALLELE

rs1127215	*PTGFRN*	1:117532790	T	C

rs703174	*/*	3:157493146	T	C

rs10937625	*STK32B*	4:5128159	C	T

rs17590046	*PPARGC1A*	4:24362541	C	T

rs28562175	*LOC105379011*	5:67827456	T	C

rs10812774	*LINGO2*	9:28294231	C	T

rs1412229	*LINGO2*	9:28478305	T	A

rs7033345	*LINGO2*	9:28717573	G	T

rs12764057	*CTNNA3*	10:68845715	G	T

rs10822974	*CTNNA3*	10:68850419	G	A

rs7903491	*CTNNA3*	10:68917164	A	G

rs3794087	*SLC1A2*	11:35329615	T	G

rs9652490	*LINGO1*	15:77963887	G	A

rs11856808	*LINGO1*	15:77972770	T	C

rs1945016	*MIR924HG*	18:37207175	G	T

rs9980363	*LINC00323*	21:42520134	C	T


/: no related data.

### Statistical analyses

The aggregate burden of rare deleterious variants of ET-associated genes between LOPD and controls was calculated using the optimized sequence kernel association test (SKAT-O) [29]. Covariates including the gender, age, and first five principal components (PCs) of ancestry were used to adjust the analyses. We first performed SKAT-O analysis on the variants of complete ET gene sets and then on the variants for each gene. Individual logistic regression analyses (PLINK v1.90) were run for each of the selected ET-GWAS related SNPs, with PD status modeled as a function of SNP allele count (0,1, or 2), gender, age, and first five PCs of ancestry, and their corresponding odds ratios (OR) and 95% confidence intervals (CI) were calculated. Significant P values (< 0.05) generated were further adjusted following the Bonferroni procedure. The corresponding Bonferroni-corrected significance threshold of P was 0.0015 for gene-based burden tests and 0.0038 for single variant tests. The data were presented as mean ± standard deviation for continuous variables and counts for categorical variables.

To estimate statistical power of the burden of rare deleterious variants of ET-associated genes, we performed SKAT simulations for the included gene set or a single gene in our cohort [30]. Related parameters were set as follows: sample size (1962 *vs*. 1279), PD prevalence (0.0043), sum of gene-set coding region lengths (146kb), average gene-coding length (4.4kb), MAF cut-off for causal variants (0.03), and penetrance (100%). To estimate the statistical power for selected ET-GWAS-related SNPs, we used GAS power calculator to assess the statistical power of our cohort [31]. Related parameters were set for each SNP: sample size (1962 *vs*. 1279), PD prevalence (0.0043), significance level (0.05), disease allele frequency, and genotype relative risk.

## Results

1962 sporadic LOPD patients and 1279 race-matched healthy controls were included in our case-control study and sequenced using WGS technology. The average sequencing depth of 12-fold and 95.5% of genome regions of at least a 5-fold coverage met our analysis. A standard bioinformatic pipeline was performed for detecting variants in the WGS sequencing data. The results of the SKAT simulations showed that our sample size could guarantee a statistical power of 100% for the gene set. We estimated an average power of nearly 47% to discover an association for single loci. Assessment of statistical power showed that our sample size could reach reliable power (>70%) for most of the selected SNPs (9/13). Four SNPs (rs1127215, rs10812774, rs7903491, and rs9980363) were insufficiently powered (32.8%~64.1%) mainly because of the relatively low value of genotype relative risk. The evaluation of statistical power indicated that our cohort was suitable for further analysis.

Further analysis for the selected set of ET-associated genes/loci, which included 33 genes and loci, identified 1453 variants in the exons and exon-intron boundaries with the minor allele frequencies (MAF) < 1% in our cohort. Logistic regression analysis, adjusted for age and sex, was used to test for association between genotype and PD. Based on our analysis strategy, variants of complete ET gene sets and the variants for each gene were analyzed by SKAT-O separately. However, there was no association between the ET gene set and the LOPD cohort within different combined categories (Table 2). In gene-based burden analysis of the ET single gene, we also observed no significant association between any gene and PD (Table 3).

**Table 2:** Analysis of ET associated genes rare variant burden in Parkinson’s disease.


CASE (n)	CONTROL (n)	VARIANTS GROUP	(a) MAF < 1%		(b) MAF < 3%	
	
			N	*P*	N	*P*

1962	1279	All	1466	0.476	1494	0.262

		LoF	30	0.415	30	0.415

		Missense	898	0.317	911	0.295

		Dmis	288	0.472	294	0.383

		LoF + Dmis	318	0.442	324	0.359


MAF = minor allele frequency; LoF = Loss of function; Dmis = Damaging missense (ReVe > 0.7); *P* value was calculated by SKAT-O (Sequence Kernel Association Test-Optimal).

**Table 3:** Analysis of ET associated genes rare (MAF < 0.01) damaging variant burden in cohort WGS.


GENES	LoF + DMIS		LoF		Dmis	
		
	n	*P*	n	*P*	n	*P*

*DRD3*	1	0.467	–	–	1	0.467

*HS1BP3*	5	0.246	–	–	5	0.246

*FUS*	8	1.000	2	0.384	6	1.000

*HTRA2*	4	0.319	2	0.327	2	0.313

*TENM4*	56	0.075	1	0.412	55	0.075

*SCN4A*	36	0.111	–	–	36	0.111

*SORT1*	5	0.659	1	0.477	4	0.614

*NOS3*	11	0.063	2	0.522	9	0.055

*KCNS2*	4	0.240	1	0.173	3	0.053

*HAPLN4*	1	0.184	1	0.184	–	–

*USP46*	1	0.318	–	–	1	0.318

*SCN11A*	14	0.703	3	0.738	11	0.353

*CACNA1G*	16	0.007	1	0.137	15	0.025

*SLIT3*	29	0.494	–	–	29	0.494

*KARS*	8	1.000	–	–	8	1.000

*KIF5A*	6	0.217	–	–	6	0.217

*NTRK1*	21	0.575	3	0.153	18	0.624

*MTHFR*	17	0.183	2	0.545	15	0.177

*LINGO1*	2	0.458	–	–	2	0.458

*LINGO2*	7	0.655	–	–	7	0.655

*MAPT*	3	0.556	3	0.556	–	–

*SLC1A2*	2	0.519	–	–	2	0.519

*HMOX1*	3	0.603	–	–	3	0.603

*HMOX2*	2	0.168	–	–	2	0.168

*TREM2*	3	0.214	2	0.330	1	0.365

*STK32B*	7	0.418	2	0.379	5	0.427

*PPARGC1A*	6	0.275	–	–	6	0.275

*CTNNA3*	25	0.444	4	0.617	21	0.499

*ALAD*	5	0.042	–	–	5	0.042

*RIT2*	1	0.500	–	–	1	0.500


*P* value was calculated by SKAT-O (SNP-set (Sequence) Kernel Association Test–Optimal.

For SNPs identified in previous ET-GWAS, three variants (rs28562175, rs1412229, and rs1945016) were excluded for further analysis because of deviated Hardy-Weinberg equilibrium in our cohort. Nevertheless, no significant association was detected between the remaining 13 SNPs and LOPD (Table 4).

**Table 4:** Association analysis of SNPs identified in the WGS cohort.


SNP	GENE/LOCUS	POSITION (hg19)	MINOR ALLELE	MAJOR ALLELE	CASE (HOM/HET/WILD)	CONTROL (HOM/HET/WILD)	MAF_CASE	MAF_CONTROL	P VALUE	OR (95%CI)	H-W P-VALUE (CONTROL)

rs1127215	*PTGFRN*	1:117532790	T	C	119/578/1088	75/359/730	0.2286	0.2186	0.4124	1.052 (0.9316–1.188)	0.001098

rs703174	/	3:157493146	T	C	43/385/1381	26/251/904	0.1302	0.1283	0.6347	1.038 (0.8911–1.208)	0.09052

rs10937625	*STK32B*	4:5128159	C	T	32/382/1427	28/266/885	0.1211	0.1366	0.135	0.8899 (0.7636–1.037)	0.1383

rs17590046	*PPARGC1A*	4:24362541	C	T	19/271/1566	10/168/1035	0.08324	0.07749	0.5715	1.056 (0.8751–1.274)	0.3103

rs10812774	*LINGO2*	9:28294231	C	T	376/736/610	225/505/384	0.4321	0.4286	0.9208	1.005 (0.9064–1.115)	0.01435

rs7033345	*LINGO2*	9:28717573	G	T	146/628/971	90/383/665	0.2636	0.2474	0.1812	1.084 (0.9631–1.22)	0.00142

rs12764057	*CTNNA3*	10:68845715	G	T	357/804/565	256/503/348	0.4397	0.4584	0.1715	0.9293 (0.8366–1.032)	0.005329

rs10822974	*CTNNA3*	10:68850419	G	A	372/774/597	232/511/400	0.4355	0.4265	0.5919	1.029 (0.9279–1.14)	0.003701

rs7903491	*CTNNA3*	10:68917164	T	G	81/491/1220	59/367/757	0.1822	0.205	0.08022	0.8917 (0.7843–1.014)	0.1078

rs3794087	*SLC1A2*	11:35329615	T	G	71/558/1221	51/361/784	0.1892	0.1936	0.9166	0.9931 (0.8716–1.131)	0.2659

rs9652490	*LINGO1*	15:77963887	G	A	197/736/867	125/502/541	0.3139	0.3219	0.5797	0.9691 (0.8672–1.083)	0.5922

rs11856808	*LINGO1*	15:77972770	T	C	0/28/1900	1/15/1232	0.007261	0.006811	0.7902	1.085 (0.5956–1.976)	0.05337

rs9980363	*LINC00323*	21:42520134	C	T	14/274/1565	8/184/1009	0.08149	0.08326	0.8135	0.9776 (0.8102–1.18)	1


/: no related data.

## Discussion

The etiology of Parkinson’s disease remains unclear, with one of the consensuses that both environment and genetics contribute to its pathogenesis, especially for the most common late-onset sporadic form of PD. Accumulating evidence demonstrated genetic factors were involved in the pathogenesis of this disorder [32,33,34]. Some research also suggested the possible genetic link between ET and PD [26,35,36]. However, there was hardly a genomics-based large-scale study exploring this topic. In our recently published paper, we have systematically investigated the association between rare coding variants in ET-associated genes and EOPD using WES data and found a suggestive association between *TENM4* and EOPD from mainland China [15]. However, EOPD represents only a small proportion of all PD cases. In the current research, we performed WGS in the 1962 sporadic LOPD cases and 1279 controls from mainland China and explored the potential genetic interplay between ET-associated genetic loci/variants and LOPD using logistic regression analysis and SKAT-O. As a result, we did not observe a significant association between the included SNPs with LOPD. Also, we did not discover a significant burden of rare deleterious variants tested of ET-associated gene in association with LOPD risk. Notably, most ET has a positive family history which indicates it is a familial disorder. A number of causative genes were reported in study of ET pedigrees. Function change of coding protein was considered to contribute to its pathogenesis [37]. However, significance of non-coding variants of these genes was unclear. Evaluation of non-coding variants contribution might be needed in our future study. Our results do not support the role of ET-associated genetic variants in LOPD.

Progress in genetic research has been made as to genetic loci and genes that confer susceptibility to ET in the past decade [38,39]. Many putative ET loci have been reported; however, most have conflicting replication results. Meta-analyses revealed only a marginal association for STK32B rs10937625 and LINGO1 rs9652490 with ET [38]. Several variants have also been examined for their possible association with ET in the Chinese population. STK32B (rs10937625) and CTNNA3 (rs7903491) were associated with ET in China in two studies [40,41]. Most association studies were reported negative results [42,43,44,45,46,47]. Meantime, some studies focusing on the genetic association between ET and PD have also emerged. Although evidence indicates that ET and PD may share genetic risk factors [26,35,36], some results did not support the link [48,49,50]. Rare deleterious variants in ET-associated genes were seldom found in Mendelian inherited PD. As to the SNPs identified in ET-GWAS, similar results were obtained in a recent association study [51], which concluded that the 22 variants identified by the ET-GWAS study were not significantly associated with PD in European population.

Conflicting results from the association between ET and PD perhaps were related to the fact that currently, we have limited knowledge regarding the cause of these two disorders. Although a number of genes were reported as the potential causative genes in ET, the conflicting results and the lack of replication for many candidate genes may suggest the listed loci or genes here were not literally the ET-associated genetic factor. Although the present study does not support a role for genetic variants of ET in LOPD, further study is required to understand the potential link between PD and ET.

### Limitations

This study has several limitations. First, estimated average statistical power for single-gene loci was only 47%. The rare variant number was mainly responsible for the poor power of the discovery of risk alleles at single loci. However, our sample size guaranteed a statistical power of 100% for the gene set, which may serve as a reliable supplement. Second, detailed information about tremor symptoms and the proportion of ET-converted PD were unavailable in our LOPD cohort, which prevented us from evaluating the underlying impact on the results. Moreover, some cases may not be PD along with a longer length of follow-up despite the fact that only 35 patients (~1.8%) who changed the diagnosis in our two-year follow up study were excluded from our cohort before initiating the analysis. However, we believe that the amount was very small and the impact was limited.

## Additional Files

The additional files for this article can be found as follows:

10.5334/tohm.885.s1 Supplementary Table 1. Summary of loci and genes associated with ET selected in our study. OR and p-values were presented for the SNPs identified in case-control studies or GWAS.

10.5334/tohm.885.s2 Supplementary Table 2. Cohort clinical and demographic data.
