# Supplementary material for: Association Analysis of Essential Tremor-Associated Genetic Variants in Sporadic Late-Onset Parkinson’s Disease
Source: Tremor Other Hyperkinet Mov (N Y). 2024 May 10;14:25. doi: 10.5334/tohm.885 (PMC11086585; doi:10.5334/tohm.885)
Supplement: Supplementary Table 1. — Summary of loci and genes associated with ET selected in our study. OR and p-values were presented for the SNPs identified in case-control studies or GWAS. [file tohm-14-1-885-s1.pdf]

Supplementary Table 1. Summary of loci and genes associated with ET selected in our study. OR and p-values were presented for the SNPs identified in case-control studies or GWAS.

Gene	Genomic location	Year of identification	OR (95%CI)	P value
DRD3	chr3q13	2006	/	/
HS1BP3	chr2p24.1	2005	/	/
FUS	chr16p11.2	2012	/	/
HTRA2	chr2p13.1	2014	/	/
TENM4	chr11q14.1	2015	/	/
SCN4A	chr17q23.3	2015	/	/
SORT1	chr1p13.3	2015	/	/
NOS3	chr7q36.1	2016	/	/
KCNS2	chr8q22.2	2016	/	/
HAPLN4	chr19p13.11	2016	/	/
USP46	chr4q12	2016	/	/
SCN11A	chr3p22.2	2017	/	/
CACNA1G	chr17q21.33	2019	/	/
SLIT3	chr5q34-q35.1	2019	/	/
KARS	chr16q23.1	2019	/	/
KIF5A	chr12q13.3	2019	/	/
NTRK1	chr1q23.1	2019	/	/
MTHFR (T677T)	chr1p36.22	2004	3.05 (1.50, 6.23)	0.001
MTHFR (T677T/A1298A)	chr1p36.22	2004	3.15 (1.51, 6.58)	0.001
MTHFR (C677C/C1298C)	chr1p36.22	2004	2.29 (0.99, 5.30)	0.047
LINGO1 (rs9652490)	chr15q24.3	2009	1.63 (1.35, 1.97)	$3.0*10^{-7}$
LINGO2 (rs1412229)	chr9p21.2-p21.1	2010	0.72(0.55, 0.94)	0.015
MAPT (rs1052553)	chr17q21.31	2011	1.32 (1.03, 1.67)	0.027
SLC1A2 (rs3794087)	chr11p13	2012	1.59 (1.38, 1.84)	$3.4*10^{-10}$
HMOX1 (rs2071746)	chr22q12.3	2015	0.64 (0.41, 0.99)	0.033
HMOX2 (rs1051308)	chr16p13.3	2015	1.44 (1.04, 1.99)	0.022
TREM2 (rs75932628)	chr6p21.1	2015	5.97(1.20, 29.63)	0.042
STK32B (rs10937625)	chr4p16.2	2016	0.80 (0.74–0.87)	$5.2*10^{-10}$
PPARGC1A (rs17590046)	chr4p15.2	2016	0.79 (0.72–0.86)	$1.4*10^{-9}$
CTNNA3 (rs12764057)	chr10q21.3	2016	1.17 (1.09–1.26)	$1.2*10^{-8}$
CTNNA3 (rs10822974)	chr10q21.3	2016	1.16 (1.08–1.24)	$1.7*10^{-7}$
CTNNA3 (rs7903491)	chr10q21.3	2016	1.10 (1.03–1.18)	$2.5*10^{-7}$
ALAD (allele 2)	chr9q32	2017	80.29(3.08,2,096.36)	0.008
RIT2 (rs12456492)	chr18q12.3	2017	1.37(1.11-1.70)	0.003
MC1R (rs34090186)	chr16q24.3	2018	2.79(1.08, 7.18)	0.027
VDR (rs2228570)	chr12q13.11	2018	2.21 (1.05–4.64)	0.033
IL1B (rs1143633)	chr2q14.1	2019	2.57(1.38-4.81)	0.003

/: inapplicable.
