# Supplementary material for: Association Analysis of Essential Tremor-Associated Genetic Variants in Sporadic Late-Onset Parkinson’s Disease
Source: Tremor Other Hyperkinet Mov (N Y). 2024 May 10;14:25. doi: 10.5334/tohm.885 (PMC11086585; doi:10.5334/tohm.885)
Supplement: Supplementary Table 2. — Cohort clinical and demographic data. [file tohm-14-1-885-s2.pdf]

Supplementary Table 2 Cohort clinical and demographic data.

**cases**				**controls**		
n	male	recruitment age (SD)	PD onset age (SD)	n	male	recruitment age (SD)
1962	50.15%	66.76 (7.08)	61.88 (6.93)	1279	47.93%	62.32 (7.11)
